# Shedding Light on the Nature of Seminal Round Cells

**DOI:** 10.1371/journal.pone.0151640

**Published:** 2016-03-16

**Authors:** Gianpiero D. Palermo, Queenie V. Neri, Tyler Cozzubbo, Stephanie Cheung, Nigel Pereira, Zev Rosenwaks

**Affiliations:** Ronald O. Perelman and Claudia Cohen Center for Reproductive Medicine, Weill Cornell Medicine, New York, New York, United States of America; University of Hyderabad, INDIA

## Abstract

**Introduction:**

In this investigation we assess the incidence of round cells (RCs) in semen samples in our infertile patient population and their significance on intracytoplasmic sperm injection (ICSI) cycle outcomes. We also evaluate the usefulness of RCs as indicators of bacterial infection and highlight the origin of this cell-type, as well as its role in the human ejaculate.

**Patients and Methods:**

In a prospective fashion, a total of 4,810 ejaculated samples were included in the study during a period of 24 months. RCs were characterized for white blood cell (WBC) components versus exfoliated germ cells by testing for multiple markers of ploidy as well as protamine assays. Cases displaying ≥ 2 x 10^6^/ml RCs were screened for bacteria. Raw specimens containing RC were processed by peroxidase and other leukocyte assays, specific stains for protamines were used to identify spermiogenic stage, aneuploidy (FISH) assessment was carried out, and the presence of various Sertoli-cell cytoplasmic remnants was analyzed to identify and characterize immature germ cells. The effect of RC on clinical outcome was assessed in specimens used for ICSI.

**Results:**

The average age of the men involved was 39.2 ± 7 years. Semen samples had a mean concentration of 40.7 ± 31 x 10^6^/ml, motility of 42.6 ± 35%, and morphology of 2.3 ± 2%. RCs were identified in 261 specimens, representing a proportion of 5.4%. Men with RCs had comparable age but lower sperm concentration and morphology than the control group (*P*<0.001). The aneuploidy rate of 4.3% in RCs group was remarkably higher than the control group (2.3%; *P*<0.001). Sperm aneuploidy rate positively correlated with the number of RCs (*P*<0.001). Of 44 men, 17 of them in 18 cycles had up to 1.9 x 10^6^/ml RCs without affecting fertilization and clinical pregnancy rates when compared to controls (n = 365 cycles). In 27 men undergoing 33 ICSI cycles with ≥ 2 x 10^6^/ml RCs, the fertilization rate trended lower and the miscarriage rate was significantly increased (*P* = 0.05). There was lack of correlation between RC and bacteriological growth. Specific markers indicated that seminal RCs are mostly immature germ cells encased in the remnants of Sertoli cell cytoplasm. Moreover, their modest protamine content and their haploid status confirm that they are post-meiotic. Sequential observation in the same man showed that RC episodes were followed by an amelioration of semen parameters, and interestingly, the episodic occurrence of RCs often coincides with flu season peaks.

**Conclusions:**

Seminal RCs are not a marker of infectiousness but rather a transient indicator of spermatogenic insult that possibly occurs in most men following a mild and transient ailment such as the flu.

## Introduction

During the past few decades there has been an increased incidence of sexually transmitted diseases (STDs) particularly in young couples aged 14–24 [1]. The long-term consequences of STDs are well established in both men and women. In men, they cause inflammation of the testicular tissue, epididymis, prostate, and seminal vesicles. While the risk of transmitting STDs to the female partner through unprotected intercourse is well known, there is growing concern that STDs or its infectious sequelae may be transmitted to the female partner during assisted reproductive techniques (ART). This possibly could occur during intrauterine insemination (IUI) during which the protective barrier represented by cervical mucus is bypassed or when oocytes are incubated with sperm during in vitro fertilization (IVF), or when the ooplasm of oocytes are injected with sperm during intracytoplasmic sperm injection (ICSI) [2].

Whether inflammation in various parts of the male genital tract causes subfertility or infertility is still debated [3]. A leukocyte count as high as 30% of the total amount of round cells has been observed in some studies of infertile men, even in the absence of inflammation or seminal bacterial infection [4]. Although there are many published studies linking the presence of inflammatory cells with compromised fertilization capacity [5–9], some have not seen any effect of leukocytospermia on fertilization and pregnancy with either conventional insemination or ICSI [10]. Others have suggested that leukocyte counts <1 x 10^6^/ml are associated with increased fertilization and pregnancy rates, suggesting a "Good Samaritan-like" effect [5,11].

In our practice we screen all patients for blood-borne pathogens and routinely culture semen specimens prior to cryopreservation. However, samples from intimate partners used in IUI or IVF are not screened except by heuristic analysis: color, visualization of bacilli, or presence of round cells (RCs). The latter, in particular, has been considered a reliable indicator of inflammation and perhaps infection within the male genital tract. When performing a semen analysis, RCs can be recognized by their shape and the lack of the quintessential characteristics of spermatozoa i.e., the typical head, mid-piece, tail components and the unique acrosome development of the spermatid [12]. Many practitioners consider RCs to be synonymous with leukocytes, though they inevitably also include immature germ cells [11,13]. Given the putative link of RCs with infection, the primary objective of this study is to evaluate the impact of seminal RCs on ICSI cycle outcomes. As a secondary objective, we also evaluate the utility of RCs as an indicator of bacterial infection, thereby shedding light on the origin of this cell-type and its role in the human ejaculate.

## Patients and Methods

### Patient and semen characteristics

This study was conducted in accordance with the research protocol approved by the Committee of Human Rights Research, Weill Cornell Medical College (WCMC). Patients undergoing ICSI agreed with the proceedings elucidated in the Clinical Informed Consent for Assisted Fertilization devised at the Ronald O. Perelman and Claudia Cohen Center for Reproductive Medicine (CRM), WCMC. All patients provided written consent for ICSI treatment as well as additional sperm testing as outlined by the Additional Male Infertility Testing protocol (WCMC, IRB 1006011085). Once written consent was obtained for both ICSI and additional testing, all patients received a copy of the consent and a contemporaneous note was made in the patient's medical record.

In a prospective fashion, a total of 4,051 men undergoing male infertility screening at our center during a period of 24 months were included in various studies, including the current report. Semen specimens from consenting men were processed according to the WHO criteria [14]. Semen samples were collected by masturbation with 2–3 days of abstinence. Samples were allowed to liquefy for at least 20 minutes at 37°C before analysis [15]. Sperm concentration and motility were assessed in a sperm counting chamber. Morphology classification was performed according to the most recent criteria [14]. RCs were identified and their number calculated. When thresholds of 1 and ≥ 2 x 10^6^/ml were reached, specimens were assessed for bacterial culture and round cell characterization.

### Identification, quantification, and characterization of seminal round cells

RCs were first seen in the Makler chamber during semen evaluation, thereafter were stained on Testsimplets^®^ slides (Origio, Cooper Surgical, Inc., Trumbull, CT, USA) or processed by Diff-Quick^™^ stain (Microptic S.L., Barcelona, Spain) to observe nuclear characteristics. Detailed identification and characterization of the RCs were performed in the center's andrology laboratory. Granulocytes were distinguished from other cell types by LeucoScreen^™^ assays (Vitrolife Inc., Englewood, CO, USA; Fertility Technology Resources Inc., Murphy, NC, USA). Occasionally, screenings for CD45 or transmembrane glycoproteins were also carried out [16]. After quantification of the respective immature germ cells, specific stains for Sertoli-cell cytoplasmic remnants were carried out. Samples were fixed in 2% formaldehyde in phosphate buffer solution (PBS) for 15 minutes at room temperature. Fixed samples were exposed to blocking buffer (PBS/0.1% bovine serum albumin (BSA)/5% goat serum) at room temperature for 60 minutes. Immunohistochemical analysis was performed with anti-vimentin primary antibody [VI-10] (Abcam, ab20346) at 1:200 dilution incubated with goat anti-mouse IgG H&L (Alexa Fluor^®^ 488) (Abcam, ab150113) and anti-inhibin-α secondary antibody [4A2F2] (Abcam, ab47720) at 1:200 dilution incubated with goat anti-mouse IgG H&L (Alexa Fluor^®^ 488) (Abcam, ab150113). The labeled samples were counterstained with DAPI/anti-fade solution (Millipore, S7113). Negative isotype controls from mouse IgM [ICIGM] (Abcam, ab91546) were utilized to exclude any non-specific binding, identify false positives, and determine the level of background noise.

To gauge the spermatogenic stage of the immature germ cell, the protamine nuclear content was estimated using aniline blue staining. Specimens were smeared and fixed in 3% glutaraldehyde for 30 minutes at room temperature. Specimens were then exposed to 5% w/v aniline blue in PBS (pH 3.5). Stained (lysine-rich histones that will react by taking up the aniline blue stain) and unstained cells (protamine-rich nuclei, with abundant arginine and cysteine) were counted and examined under the microscope at 600x magnification [17].

### Transmission electron microscopy (TEM)

Semen samples were rinsed with PBS and centrifuged for 500 g for 10 minutes (x2). The overlying PBS was removed without disturbing the pellet and the TEM buffer added (2.5% glutaraldehyde, 4.0% formaldehyde, 0.02% picric acid in 0.1 mol/l sodium cacodylate buffer) at room temperature for 15 minutes and overnight at 4°C. They were then washed in buffer, exposed to 2% osmium tetroxide (Electron Microscopy Sciences, USA) for 1 hour, dehydrated through increasing concentrations of ethanol up to 100%, and embedded in Spurr's resin (Electron Microscopy Sciences, USA). Thick plastic sections were cut and stained with toluidine blue in borate buffer. When spermatozoa were detected, ultrathin sections were cut, stained with uranyl acetate and lead citrate, and studied under a JEOL 100S TEM. Men with recurrent analyses of their specimen at our laboratory were grouped, and concentration plotted and indexed on their round cell episode(s). In order to identify an eventual temporal distribution, we plotted the round cell manifestation for each month to identify any seasonal distribution.

### Cytogenetic analysis and DNA integrity assessment of spermatozoa and round cells

Sperm DNA fragmentation assessment was carried out by terminal deoxynucleotidyl transferase-mediated deoxyuridine triphosphate-nick labeling (TUNEL) and Sperm Chromatin Structure Assay (SCSA, from an external laboratory) on the immature germ cells and sibling spermatozoa [18]. The TUNEL assay was carried out by collecting four smears from each semen sample on glass slides and air-dried. The In Situ Cell Death Detection Kit with Fluorescein isothiocyanate (FITC; Roche Diagnostics GmbH, Mannheim, Germany) was used with modifications. Each slide was fixed with 4% formaldehyde (1 ml) in phosphate-buffered saline (PBS) solution and incubated at room temperature for 1 hour. Slides were washed with ice-cold PBS then permeabilized with TritonX in 0.1% sodium citrate for 5 minutes. Slides were again washed with PBS then incubated with a mixture of the TUNEL enzyme solution containing terminal deoxynucleotidyl transferase plus TUNEL labeling solution containing deoxyuridine triphosphate. A Parafilm M strip (Alcan Packaging, Darien, CT, USA) was applied to each slide, and the slides were incubated in a dark, moist chamber at 37°C for 1 hour. After labeling, slides were taken out of the chamber, the Parafilm M was removed, and the cells were washed with PBS. Vectashield (Vector Laboratories, Burlingame, CA, USA) with 4’,6-diamidino- 2-phenylindole (DAPI) was applied to each slide for DNA counterstaining, and a cover slip was applied. Two negative and two positive controls were tested with each batch. Slides were analyzed using an epifluorescent microscope at 1000x magnification. The number of DAPI- and FITC-positive cells in the same field were counted and recorded. At least 500 cells were counted for one single tally on a Nikon Eclipse Ni fluorescent microscope.

### Aneuploidy testing on spermatozoa

To assess the ploidy status of the immature germ cells, a modified fluorescence in situ hybridization (FISH) for 9 chromosomes was used [19,20]. Fixation of cells was performed by placing the slides in methanol and glacial acetic acid (3:1 vol/vol) for 15 minutes, after which they were dried and stored in a slide moat overnight. Sperm denaturation was performed by placing slides in a Coplin jar containing an isothermal denaturing reagent (Cellay Inc., Cambridge, MA, USA) and ethanol for 10 minutes. The slides were then transferred into jars containing 85% and 100% ethanol for 1 minute each for permeabilization of the sperm membrane. After the slides were air dried, hybridization of the immature germ cells was carried out for 5 minutes at 37°C with two sets of probes specific to chromosomes X, Y, 15, 16, 17 and 18 (Cellay Inc., Cambridge, MA, USA). The hybridized slides were washed for 5 minutes in 2x standard saline citrate (2x SSC; Sigma Chemical Co., St. Louis, MO, USA). A total of 500 cells were assessed.

To evaluate the occurrence of aneuploidy in the coexistent spermatozoa, our standard FISH assessment for chromosomes X, Y, 13, 15, 16, 17, 18, 21 and 22 was used [18]. Nuclei were counterstained with 7 μl DAPI solution (DAPI II Counterstain, Abbott Laboratories, IL, USA), cover-slipped, and assessed on an Olympus BX61 fluorescent microscope. A minimum of 1000 cells was assessed. During chromosome assessment, normal haploid sperm nuclei were those carrying only one signal for each autosome and gonosome. Sperm missing a signal were recorded as nullisomic for the corresponding chromosome, while those carrying an extra signal were recorded as disomic. A sperm cell was classified as disomic if two fluorescent signals of the same color were observed within the sperm head. The resultant FISH error was estimated at 2–3%, based on in-house studies.

### ICSI treatment and outcome evaluation

Oocyte retrieval was performed after ovarian superovulation with gonadotropins and pituitary desensitization with GnRH-agonists or antagonists [21,22]. The choice of a stimulation protocol was dependent on patient age, etiology of infertility, previous treatment history and physician preference. For all patients, one of several established stimulation protocols was utilized; Lupron down regulation, microflare Lupron or GnRH antagonist. Human chorionic gonadotropin (hCG) was administered (3,300–10,000 IU) when at least two follicles had reached ≥17-mm diameter as observed by ultrasonography. hCG dosage was tailored according to estradiol (E2) level and body mass index. Oocyte retrieval was performed approximately 35–36 hours after hCG administration via transvaginal needle guided aspiration. These oocytes were then exposed to 40 IU recombinant hyaluronidase (Cumulase^™^, Halozyme Therapeutics, Inc. San Diego, CA) to remove cumulus-corona cells in a previously defined manner [21,22]. Details of the microinjection procedure have been previously described, including selection, immobilization and permeabilization of the spermatozoa [22–24]. At all times it was necessary to substitute an 8μl drop in the injection dish with 3 μl of the final sperm suspension [18]. Oocytes were examined 12–17 hours after the injection procedure to assess for normal fertilization, defined by the presence of two distinct pronuclei (PN) and two clear polar bodies. Evaluation for embryonic cleavage was performed every 24 hours [25]. Morphologically good quality embryos were transferred into the uterine cavity on the third or fifth day after the microinjection procedure [26].

Starting on the day of oocyte retrieval, methylprednisolone (16 mg/day) and tetracycline (250 mg every 6 hours) were administered for 4 days to all patients. Progesterone administration (25–50 mg IM/day) was started the day after retrieval and was continued until the establishment of pregnancy. A serum βhCG assay was performed 14 days after the oocyte retrieval. A biochemical pregnancy was defined as a positive βhCG level that decreased prior to when an ultrasound could detect an implantation site. A clinical pregnancy was defined as the presence of at least one fetal heartbeat by ultrasound assessment during the 7th week of gestation.

Semen parameters, maternal age, fertilization rates, and pregnancy characteristics were recorded. Presence of RCs was noticed and categorized between 1- to 1.9 x 10^6^/ml or ≥ 2 x 10^6^/ml and compared to specimens void of RCs that were used as a control. To assess the impact of the coexistence of RCs only cryopreserved specimens were utilized for ICSI. Embryological outcome was analyzed in relation to the increasing concentration of RCs.

### Statistical analysis

Statistical comparison to evaluate all relevant hypotheses was carried out by χ^2^ analysis, two-tailed at 5% level of significance. Where appropriate, Fisher’s exact tests were used to ensure no violation secondary to the small cell counts in χ2 procedures. Student's *t*-test was used to compare means. Statistical differences (*P*<0.05) were recorded in text and tables only when significant. All statistical analyses were carried out using the SPSS software (SPSS Inc., Chicago, IL, USA).

## Results

### Presence of round cells and sperm characteristics

From January 2012 to December 2014, a total of 4,810 ejaculated samples were processed for semen analysis in the center's andrology laboratory. The average age of the men was 39.2 ± 7 years. Semen samples had a mean concentration of 40.7 ± 31 x 10^6^/mL, motility of 42.6 ± 35%, and morphology of 2.3 ± 2%. RCs were identified in 261 of the specimens evaluated representing a proportion of 5.4%. Specimens presenting with RCs were produced by men of the same age as the control but were characterized by a lower sperm concentration (*P*<0.001) and poorer morphology (*P*<0.001) (Table 1). This was consistent with the findings of other studies that reported a negative correlation between sperm concentration and number of RCs [27,28].

**Table 1:** Overall comparison of patients with or without seminal round cells.

No. of	No RC	RC
Patients	3,913	138
Specimens	4,549	261
Male age (M yrs ± SD)	39.3 ± 7	39.1 ± 7
Initial concentration (M x10^6^/ml ± SD)	45.6 ± 28*	35.7 ± 34*
Initial motility (M% ± SD)	43.3 ± 15	41.9 ± 20
Morphology (M% ± SD)	2.5 ± 1^†^	2.1 ± 2^†^

M = mean, SD = standard deviation

*^†^Student *t*-test, two independent samples; Effect of presence of round cells on sperm concentration and morphology, *P* = 0.0001

### RCs as an infection marker and its consequence on sperm chromatin integrity

**Fig 1:**
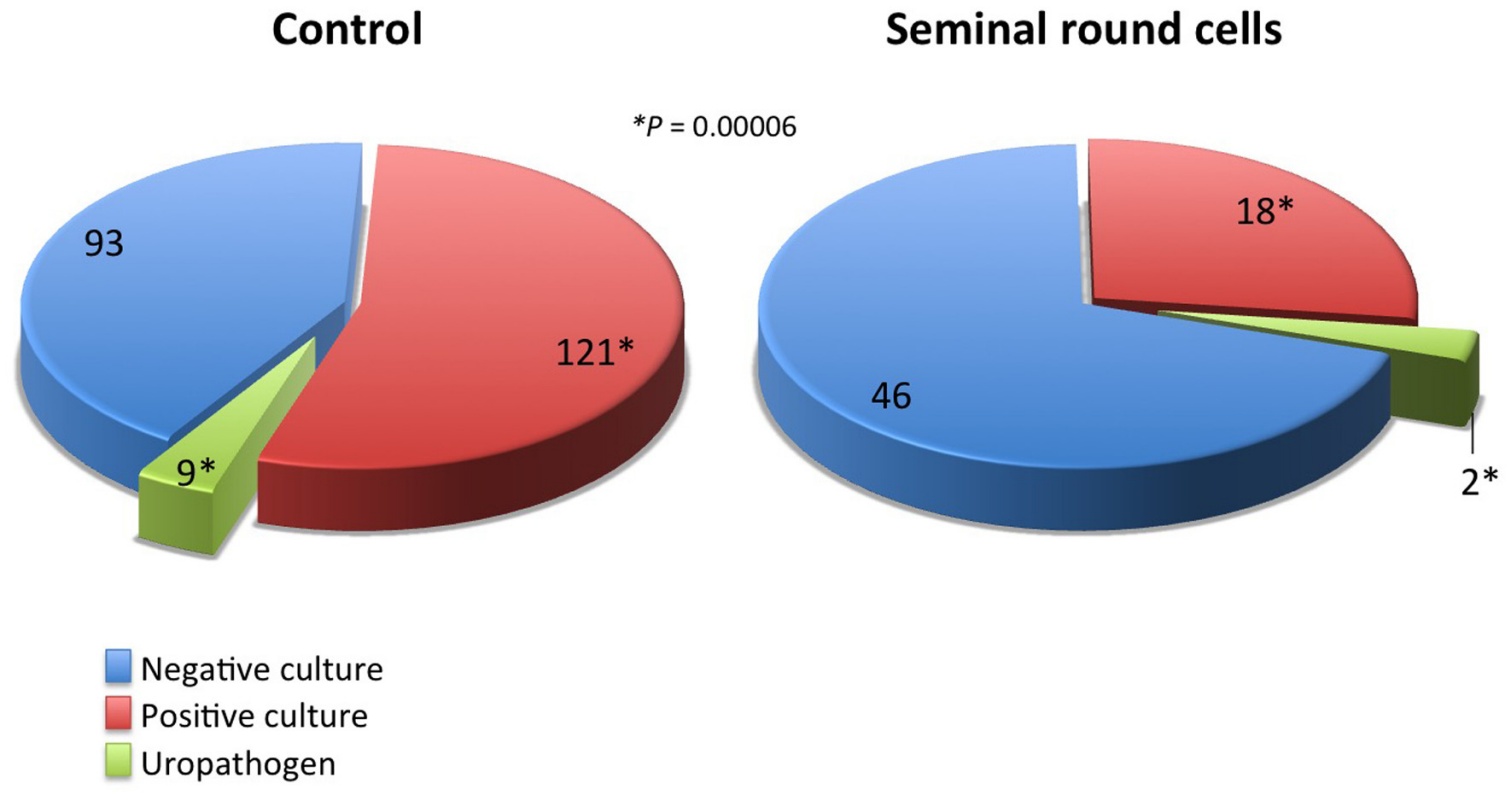
Proportion of men presenting with bacterial growth (saprophytic and pathogenic) according to presence or absence of RCs.

Presence of RCs in ejaculates has often been interpreted as a sign of inflammation [7,8, 29] or infection [30–32]. Therefore, a cohort of 289 cryopreserved samples was tested for infection status by bacteriological cultures, of which 51.9% had a positive result. Specimens with RCs had only 30.3% (20/66) (Fig 1) of positive culture results remarkably lower than the specimen serving as control (*P*<0.001). The qualification of the specific bacteria involved (whether true pathogens or saprophytes) showed that the proportion of uropathogens was equally distributed between the two groups of 7–10%. This indicated that RCs cannot function as a direct indicator of infection, as other reports have also confirmed [32–35].

We then investigated if the presumed inflammation within the male genital tract represented by the presence of RCs might affect the spermatozoa’s DNA integrity, as suggested in a previous report [30]. When specimens with similar parameters were processed for sperm chromatin fragmentation, those with RCs had a DNA fragmentation rate of 26.3%, remarkably higher than the control of 16.3% (*P*<0.001) (Table 2).

**Table 2:** Comparison of a specimen's sperm DNA fragmentation index (DFI) void of round cells versus those with round cells.

No. of	No RC	RC
Time period	Jan 2012 –Dec 2014	
Patients	224	58
Specimens	321	68
Male age (M yrs ± SD)	40.1 ± 6	39.1 ± 6
Initial concentration (M x10^6^/ml ± SD)	50.5 ± 41	44.5 ± 37
Initial motility (M% ± SD)	46.1 ± 21	46.1 ± 17
DFI (M% ± SD)	16.3 ± 12*	26.3 ± 15*

M = mean, SD = standard deviation; DFI = DNA fragmentation index (assessed by SCSA)

*Student t-test, two independent samples; Effect of presence of round cells on sperm DNA fragmentation, *P* = 0.0001

Indeed, leukocytospermia has been associated with high oxygen free radical production and increased DNA fragmentation index (DFI) [36]. This has also been confirmed in specimens with RC concentration below 1 million leukocytes [37]. The aforementioned absence of correlation between RCs and bacterial growth indicate that these cells may not serve as marker of infection or an indication for bacteriological culture on a specimen. However, one can speculate that their presence correlates with compromised chromatin integrity in the spermatozoa. This would imply that leukocytospermia may have an origin other than an infectious agent and may be a response to spermatozoa antigen resulting from a breach in the sperm-blood barrier, consequently effecting male fertility [38].

### RCs and ICSI outcome

In an attempt at quantifying the concentration of RCs in the specimens and their effect on the clinical outcome we identified 408 men whose cryopreserved samples were employed in 416 ICSI cycles carried out with their respective partners (Table 3). In 44 men, RCs were identified in 51 samples, with a concentration up to 1.9 x 10^6^/ml in 18 and ≥ 2 x 10^6^/mL in 33 cycles (Table 3).

**Table 3:** Comparing the presence and quantity of round cells with ICSI outcome (2012–2014).

		Round cell (RC) concentration (x 10^6^/ml)	
No. of (%)	Control	0 < RC < 2	≥ 2
Patients	364	17	27
Cycles	365	18	33
Female age (M yrs ± SD)	38.1 ± 5	36.9 ± 6	38.0 ± 4
Fertilization (2PN/MII injected)	2415/3206 (75.3)	102/133 (76.6)	182/262 (69.5)
Clinical pregnancy (+FHB)	131 (35.9)	11 (61.1)	12 (36.4)
Delivery and ongoing	112 (30.7)	8 (44.4)	8 (24.2)
Pregnancy loss	19 (5.2)*	3 (16.7)*	4 (12.1)*

M = mean, SD = standard deviation, 2PN = 2 pronuclei, MII = metaphase II; FHB = fetal heart beat

*χ^2^, 3 x 2, 2*df*, Effect of round cell concentration on pregnancy loss, *P* = 0.05

The average female partner age was 37.6 ± 6 years among all groups. In men with RC up to 1.9 million RCs/ml (including and exceeding the threshold for leukocytospermia required by the WHO), the fertilization rate was unaffected and the clinical pregnancy rate appeared higher. For samples with ≥ 2 million RCs, the fertilization rate instead trended lower, with a comparable pregnancy rate and a significantly higher pregnancy loss rate (*P* = 0.05). This is somewhat consistent with the findings of Barraud-Lange et al. [14] i.e., at low concentration (<1 x 10^6^/ml), leukocytospermia may be considered physiologic and seems to be associated with higher fertilization and pregnancy rates, while pregnancies resulting from spermatozoa that are associated with a higher concentration of RCs are lost more frequently.

### Seminal round cells and spermatogenic meiosis

**Fig 2:**
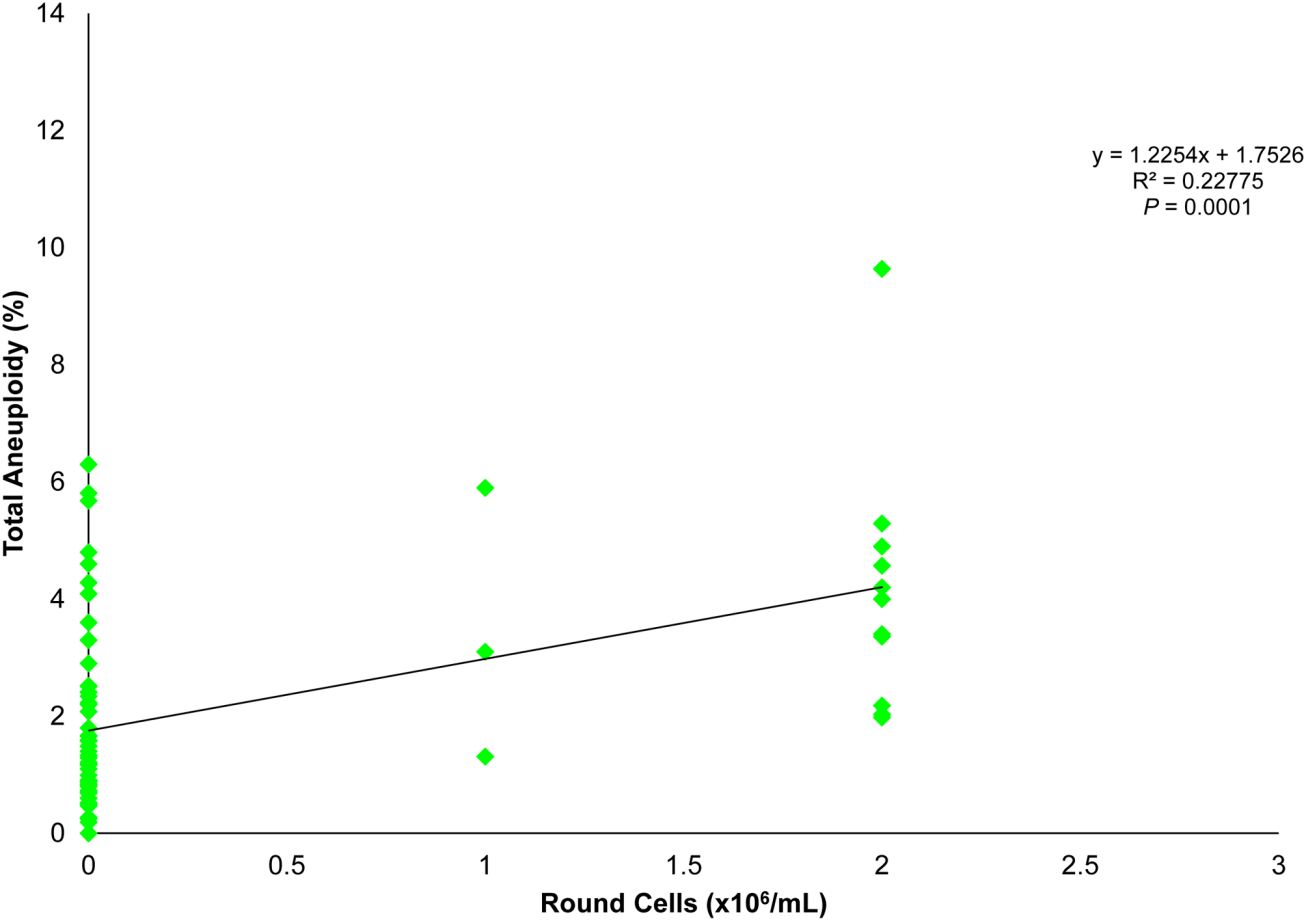
Correlation between the occurrences of aneuploid spermatozoa in relation to RC concentration in the ejaculate.

To further investigate the apparent effect of the presence of RCs on spermatozoa and consequently on pregnancy outcome, a group of men (n = 91) were screened for sperm aneuploidy. There was an occurrence of RCs of 30.8% (28/91) in these semen specimens, with a spermatozoal aneuploidy rate of 4.3%, remarkably higher than the control (2.3%; *P*<0.001). Moreover, there was a positive correlation between the aneuploidy rate and amount of RCs (*P*<0.001; Fig 2). To measure whether this higher incidence of paternal chromosomal defect associated with high RC presence would translate to compromised embryo developmental outcomes, 60 men from this cohort underwent ART treatment with their partners and 16 presented with RCs at the time of ICSI. Men with RCs had a fertilization rate of 70.3%, compared to the control group of 68.8% and clinical pregnancy rates of 31.3% and 34.1%, respectively. The pregnancy losses, however, were clearly higher (60.0% versus none in the control) in specimens containing RCs corresponding to high sperm aneuploidy (*P* = 0.01). Gonosomal disomies and disomies of chromosomes 13 and 18 were the most frequently seen meiotic abnormalities for spermatozoa isolated from specimens contaminated with RCs. This is in line with other reports that mention the paternal inheritance of aneuploidies for these specific chromosomes in the resulting embryos [39–42].

### Origin of RCs

**Fig 3:**
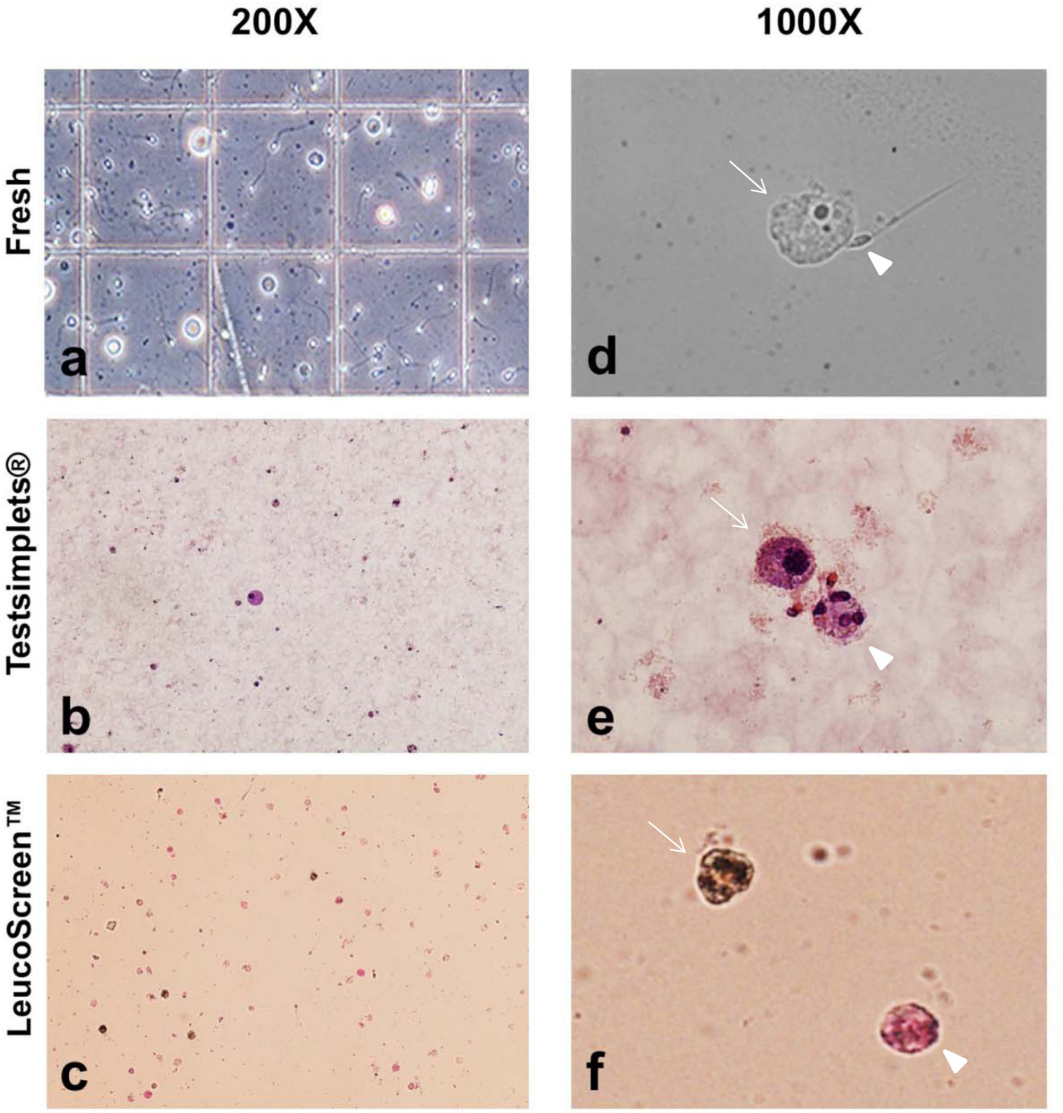
The first row are fresh specimens (a,d), the second row is Testsimplet^®^ (b,e), and the third is LeucoScreen^™^ (c,f). The left panel evidence seminal RCs assessed at 200X under phase contrast at initial semen evaluation in a counting chamber (a), following a standard morphology stain (b) and following peroxidase assay (c). In the right panel under 1000X, a fresh multinucleated RC (arrow) is visible with an adjacent spermatozoon (arrowhead) (d), stained RC (arrow) and another multinucleated RC (arrowhead) (e), and at the top is a peroxidase reactive cell (leukocyte) (arrow), and a peroxidase negative RC (arrowhead) (f).

While their presence is inconsistent and unpredictable, the origin of this poorly characterized cell-type is nevertheless a matter of great interest due to its putative effect on pregnancy outcomes [12] (Fig 3). Their presence has often been considered a manifestation of leukocytospermia, inducing many laboratories not to perform any white blood cell characterization. Conversely, specimens with increased RCs at some centers are labeled as infected and sent for bacterial culture, with consequent increased costs (and antibacterial treatment) and emotional burden for the subfertile man. It is indeed the inability to directly link RCs to infection that has induced us to quantify and characterize this putative inflammatory component. It has been observed that a portion of RCs in the semen ejaculate is represented by immature germ cells possibly round spermatids but they have been poorly characterized [12]. In our patient population the overall prevalence of semen specimens with RCs was 5.4% (261/4810), and following specific leukocyte identification and labeling, the actual fraction of white blood cells (WBC) within those specimen was at most 26.7% (172 million/644 million [WBC/RC]), with the remainder overrepresented as immature germ cells (IGC; range 0.79–24.9 million).

In a few men, a proportion of white blood cells at ≥ 50% of the seminal RC was recorded. A detailed analyses of their recent medical and surgical histories evidenced: 1) retrograde ejaculation with partial vasal obstruction in a cystic fibrosis (CF) carrier; 2) a man with chronic herpes simplex virus type 2 infection; 3) a right varicocele at ultrasound in a man injured in a contact sport; 4) an older man surgically treated for prostate cancer with a discolored ejaculate; and 5) a man who received an embolization for a grade 3 varicocele. A bacteriology assessment carried out in 4/5 of them evidenced a sperm saprophytic bacterial growth in only one instance.

**Fig 4:**
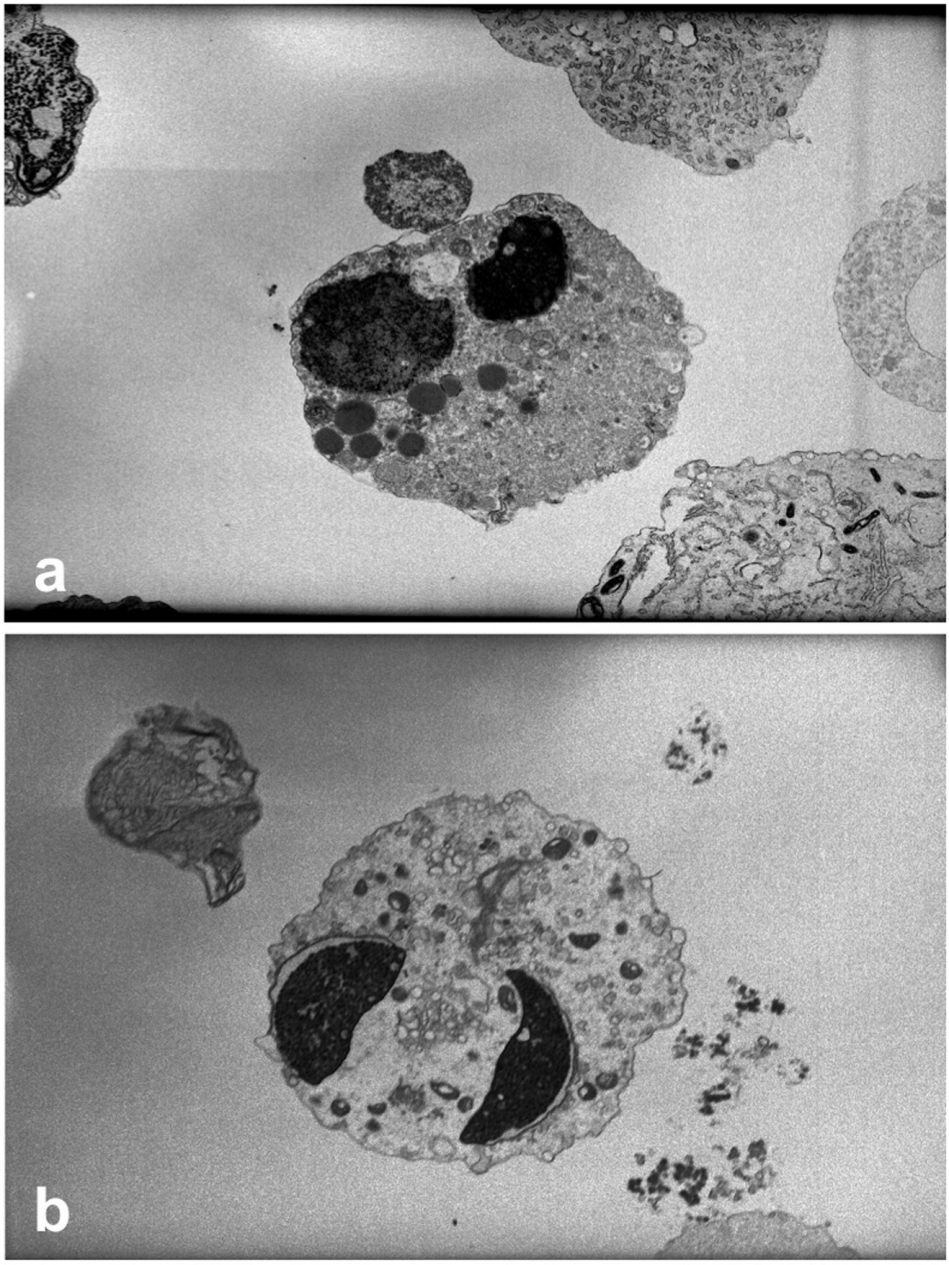
Transmission electron microscopy of multinucleated seminal round cells. Two large nuclei, round (a) and elongated (b), enclosed in poorly delineated cytoplasm sloughed off the Sertoli Cell. The vacuolar structures, particularly clear in figure (a) are verisimilar lysosomes and residual bodies (a,b).

The inconsistent size of these cells, together with the extreme fragility of their cytoplasmic membrane and the observed multinucleation without the chromatinic bridges typical of granulocytes, hinted that they may not indeed be leukocytes. Moreover, a detailed morphological microscopic observation of a stained cell indicated that the nucleus of the RC is often times multinucleated but still resembles the nucleus of the spermatozoon in shape (Fig 4a and 4b). This indeed suggests the possibility that most RCs may be immature germ cells that, having failed to complete spermiogenesis, were encased in Sertoli-cell cytoplasm that was exfoliated upon reaching the ejaculate.

**Fig 5:**
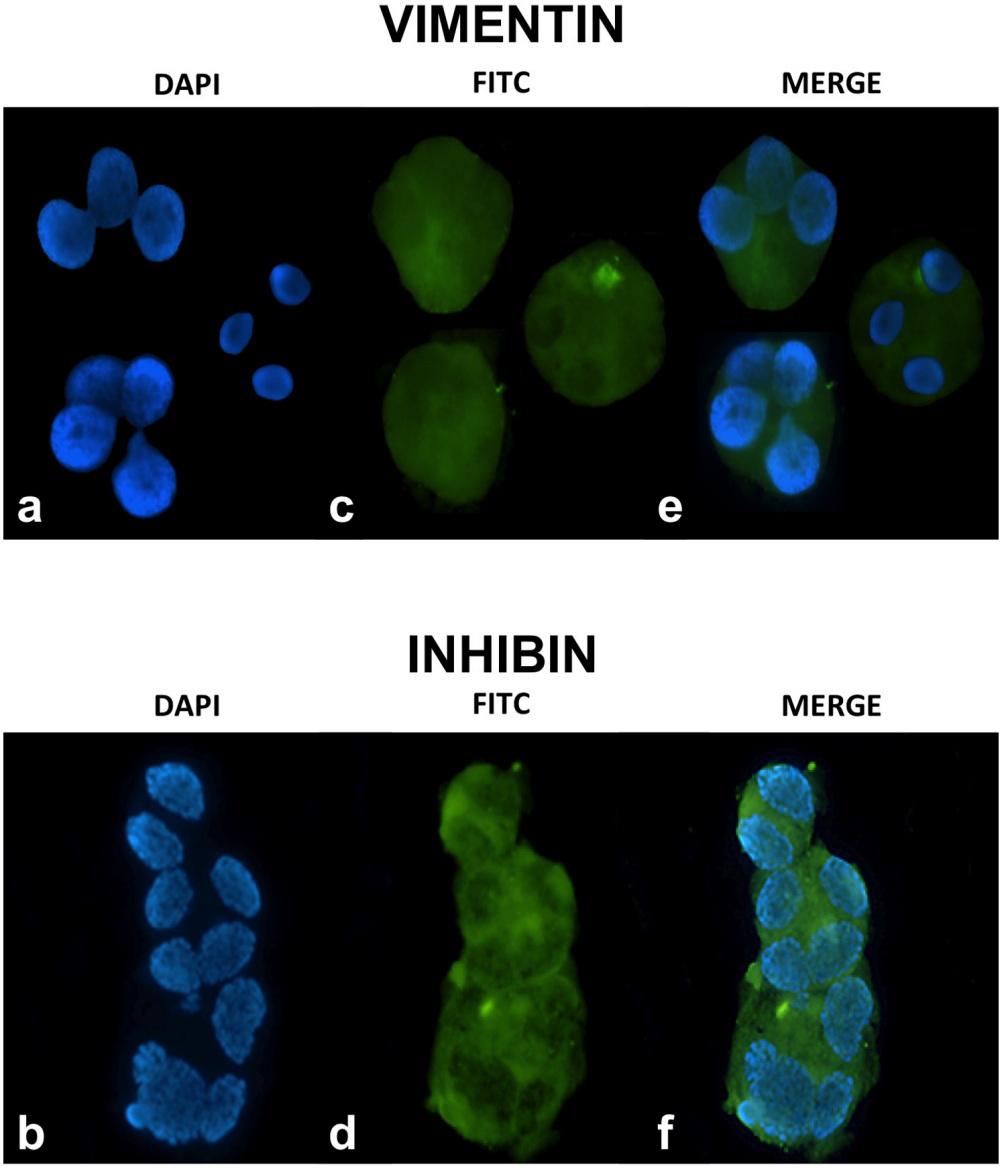
Staining of seminal RCs providing for each stain nuclear labeling (DAPI) (a, b), with markers specific for Sertoli-cell cytoplasm (vimentin, c), solely for Sertoli cell intracytoplasmic components (inhibin B, d), and merged images (e, f).

**Fig 6:**
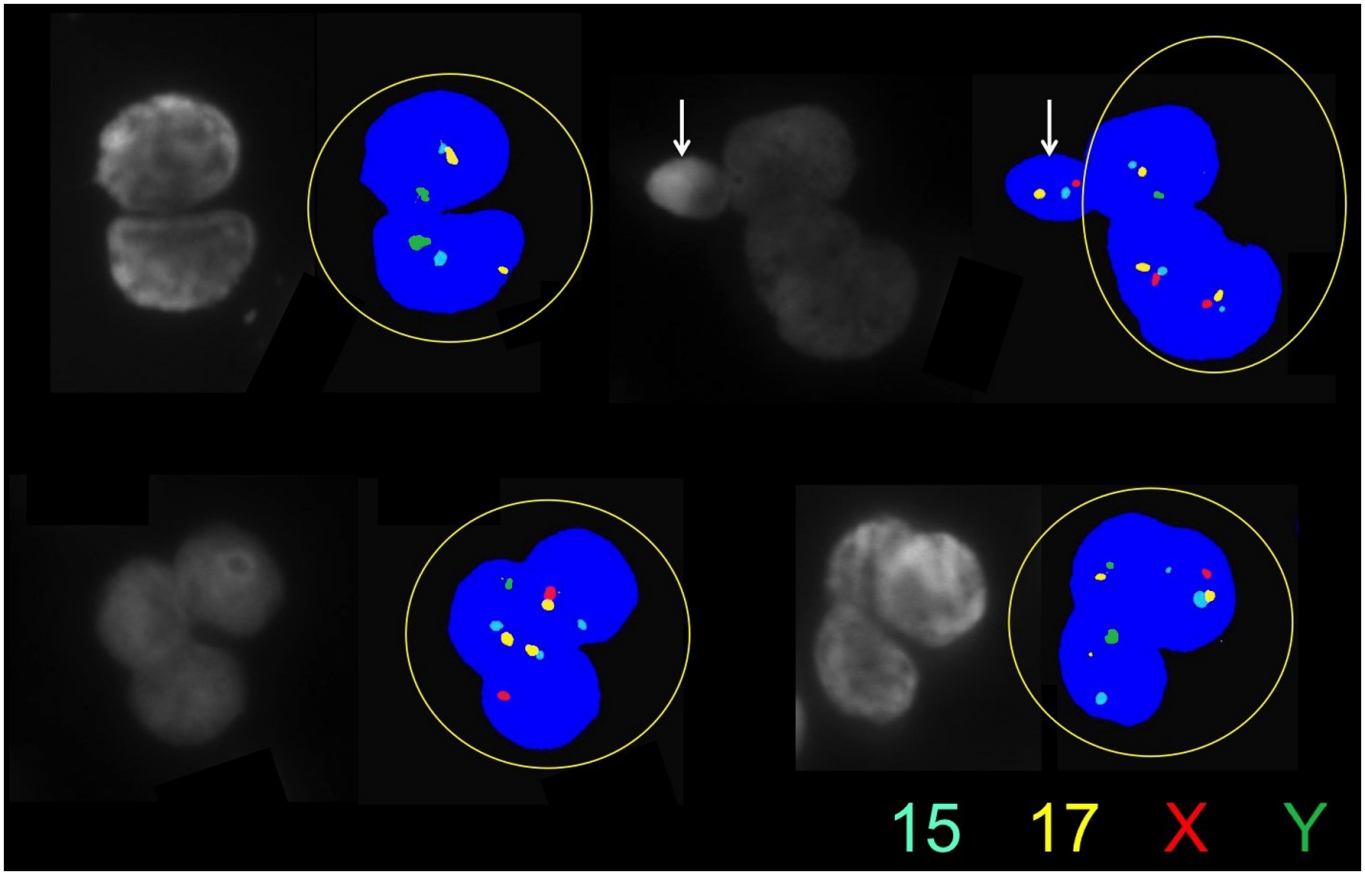
Cytogenetics of seminal multinucleated RCs carried by multicolor FISH assessing four chromosomes (X, Y, 15, 17) and confirming the haploid content of each individual nucleus. The white arrow indicates a spermatozoon.

In order to test this hypothesis, we stained RCs with vimentin, a specific marker for Sertoli cells (Fig 5a, 5c and 5e). Vimentin is VIM gene product, a protein-type III intermediate filament protein expressed in mesenchymal cells, such as the one present in the cytoplasm of Sertoli cells [43]. To rule out aspecific binding, we also utilized inhibin B, a cytoplasmic marker of Sertoli cells. We stained for the common alpha subunit of inhibin that in Sertoli cells is only represented by inhibin B (Fig 5b, 5d and 5f), a member of the TGF-b superfamily [44]. The cytoplasm of these immature germ cells stained positively for both assays (Fig 5a–5f), confirming encasement of the germ cell nuclei in Sertoli-cell cytoplasm. To further characterize their meiotic stage, we assessed the ploidy of these cells, as they all proved to be individually or multiple n-ploid chromosomes that displayed multiple nuclei, i.e., each specific nucleus had one haploid set of chromosomes, confirming their post-meiotic status (Fig 6). Interestingly, the nuclei appeared somewhat decondensed in comparison to the concurrent spermatozoa. To pinpoint the spermatogenic stage and understand the level of compaction of the chromatin, we stained for protamine presence that resulted in mildly compacted cells. This confirmed that incomplete compaction would render chromatin unstable, explaining the high DNA fragmentation detected in these RCs when compared to their respective coexistent spermatozoa (*P*<0.001).

### Round cell events and germinal epithelium output

**Fig 7:**
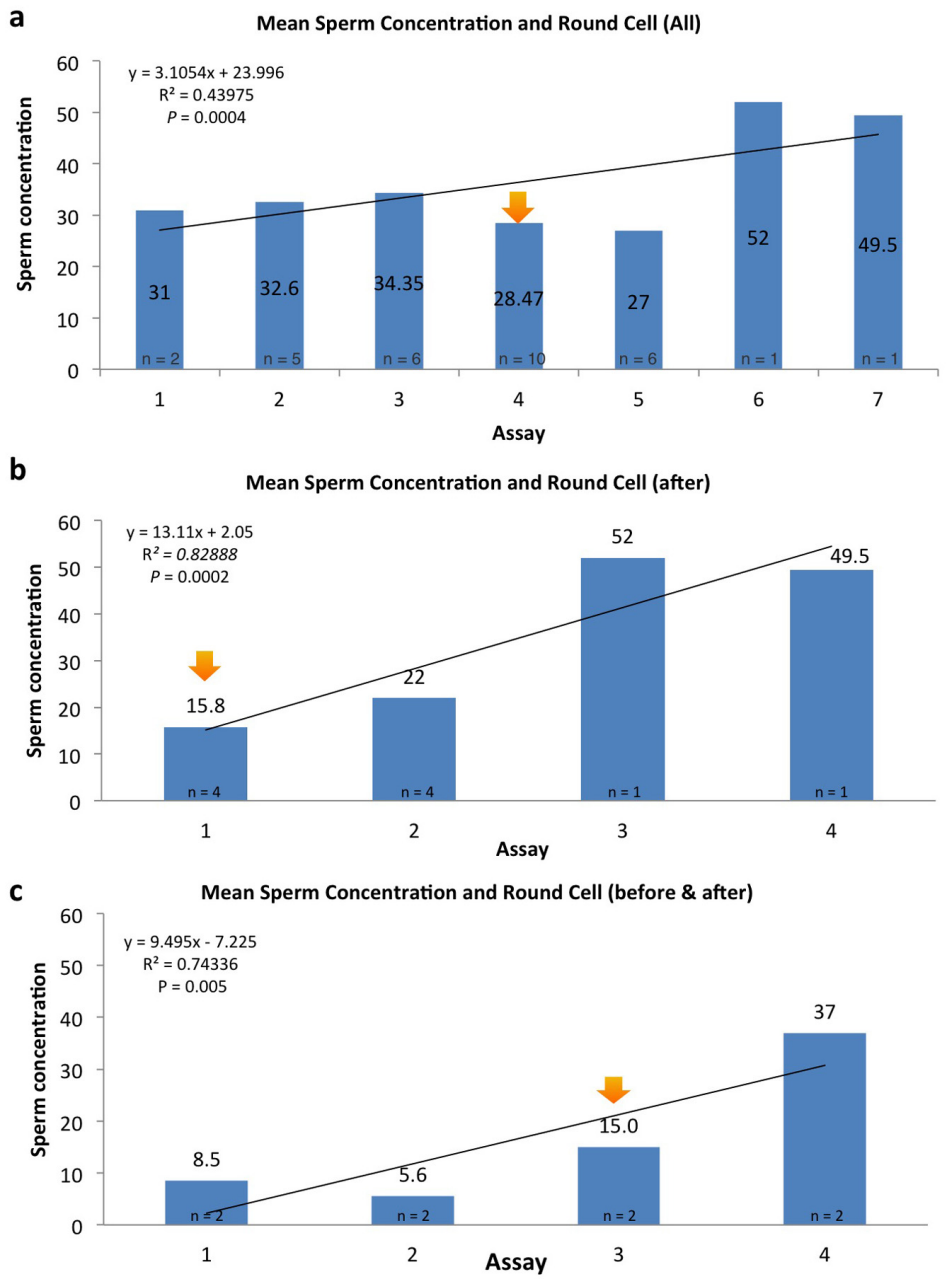
In graph (a) all men who had several semen evaluations and with one occasion of seminal RCs (orange arrow). In graph (b), we show only men who presented at first analysis with RCs (orange arrow) in their ejaculate while in graph (c), the remainder of cases present with RCs in their intermediate (orange arrow) evaluation. In all of them there is an increase in spermatozoal production following the RC episode.

**Fig 8:**
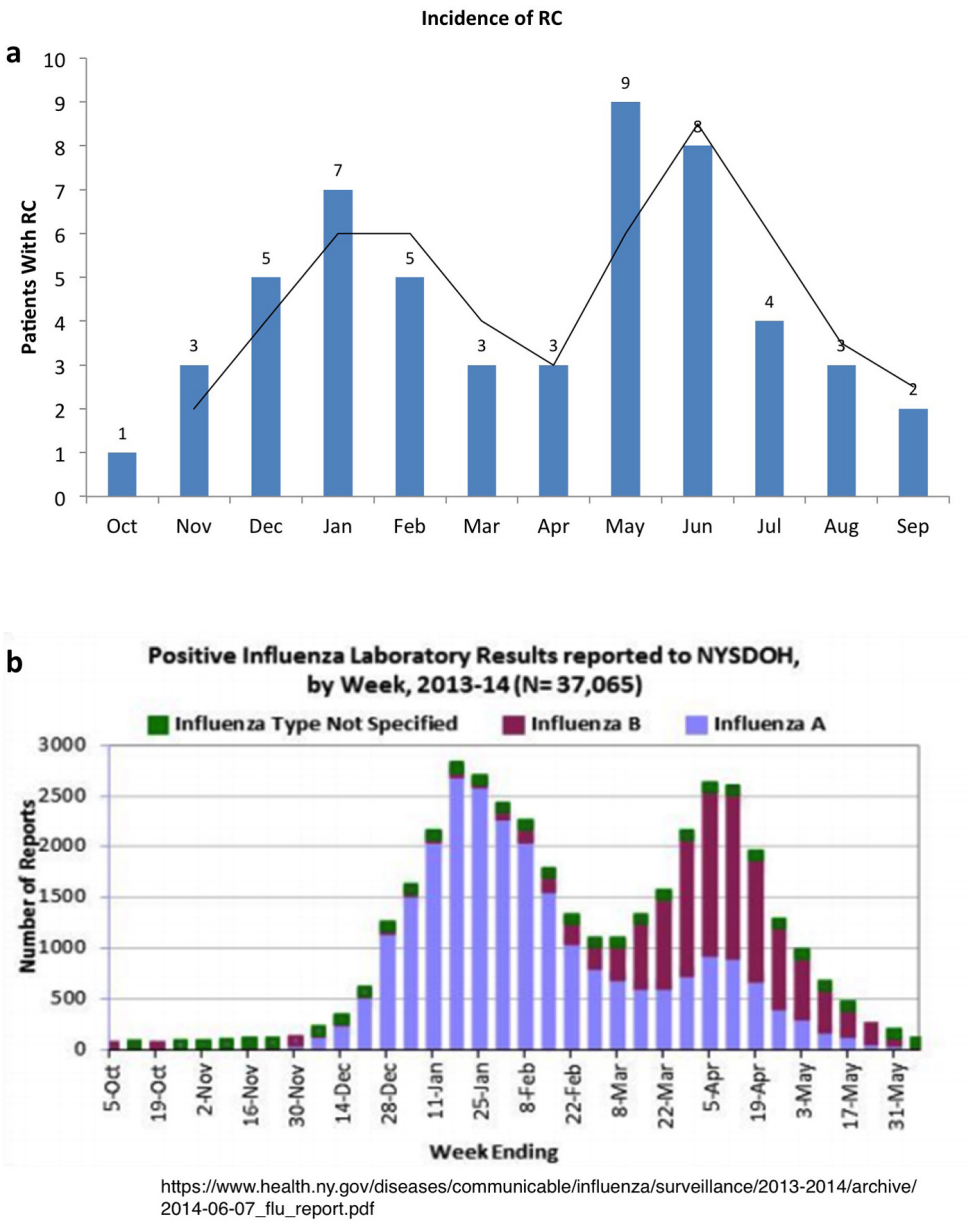
This graph (a) indicates the monthly allocation of specimens with RCs throughout the calendar year. The bimodal distribution almost exactly overlaps the Positive Influenza Laboratory Results Reported to New York State Department of Health (b).

In men that were repeatedly assessed for semen analysis at our center, we plotted the RC event in a graph by ranking their temporal spermatozoal concentration. It appeared that RC event coincided with the end of a putative insult to the germinal epithelium, as indicated by the increase in spermatozoa output following this appearance (Fig 7a–7c). To understand what the insult may be, we attempted to find patterns in the temporal occurrence of RCs and noticed that they peaked in January-February and May-June (Fig 8a). This profile nearly overlaps, with about a month's difference, with the graph published by the Influenza Outbreak Surveillance Report from New York State for the flu season 2013–2014 (Fig 8b). This suggests that the emergence of RCs might be the result of the sloughing of sperm cells that failed spermiogenic completion. This may yield nuclei that are only partially compacted, explaining the higher DNA fragmentation in RCs. This spermiogenic byproduct, while not a marker of infection of the male reproductive tract, may well be by itself responsible for the WBC component. This may be inferred by the variable proportion of WBC in contrast to the steady numbers of immature germ cells observed in men that presented with recurrent episodes of seminal RCs.

## Discussion

The assessment of the infertile man remains difficult because of the different arrays of injuries that can be exerted to the male gamete affecting its production, functional performance, and genetic makeup. The current method of screening for male infertility is characterized by a semen analysis. This test has evolved over four decades but it is still considered limited at best in its ability to predict male reproductive capacity. One of the most puzzling aspects of semen analysis is the presence of RCs. When reported at concentrations of at least one million per milliliter, as suggested by the most recent WHO guidelines, they are to be considered a sign of leukocytospermia.

With the need to understand the significance and the action to be taken when seminal RCs are present, we attempted to characterize the origin of RCs and gain insight regarding their impact on the spermatogenic process. While we believe that RCs are present in almost all specimens, the scarce evidence that the presence of RCs are linked to an acute infectious process has been confirmed by the lack of bacterial growth in the current, whether saprophytic or true uropathogens. Indeed, in the few men with an overwhelming representation of WBC within their ejaculates without acute infection or bacterial growth, the high component of polymorphonucleocytes may be explained by disruption of the blood-testis barrier after a possible systemic insult (prostate cancer, for example) that causes disarray in the germinal epithelium. The recognized proportion of WBC contained in specimens has helped us to elucidate whether a concentration of them may actually be physiologic and associated with a superior reproductive outcome [11]. In fact, the majority of RCs are immature germ cells. Their encasement in remnants of Sertoli cell cytoplasm may indicate that those cells, in spite of achieving meiotic completion as proven by their ploidy, have missed the check point that enable them to grow into a healthy spermatozoon, possibly due to failed chromatinic compaction or centrosomal acquisition that allow coupling to a flagellum. This finding, however, does not highlight the reasons for the appearance of RCs in the ejaculate, but suggests that the transient injury of the germinal epithelium that produces them is often followed by an increase in sperm production, generating the previously described "Good Samaritan-like" effect [5,11]. Moreover, their simultaneous appearance with high incidence of flu reassures us that RCs are certainly not a gauge of infection, but a marker of an active regeneration of the spermiogenic process that may affect any man throughout his spermatogenic life. The concerns about the observation of these cells, however, still linger because they signal a high turnover of the germinal epithelium within the seminiferous tubules that can be accompanied by meiotic deviations and high aneuploidy rates of the concurrent spermatozoa produced. Moreover, the source of reactive oxygen species as per their WBC contribution and even from membrane remodeling occurring during spermiogenesis such as head-tail attachment defects, incomplete acrosome development, or sperm cytoskeleton defects may compound this spermatogenic dysfunction [45–47]. It may indeed be that this high turnover of the germinal epithelium may consequently affect DNA integrity and chromosomal status of the sibling spermatozoa, as other authors have argued [36,37]. During spermatogenesis, apoptosis is a major mechanism controlled by Sertoli cells dedicated to the elimination of defective germ cells and the appearance of unpackaged nuclei wrapped in Sertoli-cell cytoplasm that may indicate an overwhelmed patrolling mechanism.

This may concurrently translate in meiotic errors resulting in reproductive inefficiency as indicated by the higher incidence of pregnancy losses at very high round cell concentrations. This study suggests that when an ejaculate manifests with RCs, they should be counted and the proportion of WBC determined. A repeated evaluation of the ejaculate at the following spermatogenic cycle will provide a more realistic assessment of the condition of germinative epithelium of these subfertile men.
